# Psychosocial impact of self-perceived malocclusion on oral health-related quality of life in Nigerian adolescents and young adults: a cross-sectional study

**DOI:** 10.1186/s12903-026-09399-9

**Published:** 2026-07-24

**Authors:** Khadijah Musa Sa’ad, Adetayo Aborisade, Bernard Ogbozor, Tope Emmanuel Adeyemi

**Affiliations:** 1 Department of Child Dental Health, Bayero University, Kano, Nigeria; 2 Department of Oral Diagnostic Sciences, Bayero University, Kano, Nigeria; 3 Department of Oral and Maxillofacial Surgery, University of Nigeria, Nsukka, Nigeria

**Keywords:** Malocclusion, Psychosocial impact, Dental aesthetics, Oral health-related quality of life, Adolescents, Nigeria

## Abstract

**Background:**

Malocclusion can negatively impact psychosocial well-being and oral health-related quality of life (OHRQoL), particularly during adolescence and young adulthood. This study evaluated the prevalence and severity of psychosocial burden associated with self-perceived malocclusion and its relationship with OHRQoL among adolescents and young adults in Kano State, Nigeria.

**Methods:**

This cross-sectional study included 316 participants aged 13–25 years recruited from secondary schools and a university in Kano metropolis using convenience sampling. Data were collected using self-administered questionnaires comprising the Aesthetic Component (AC) of the Index of Orthodontic Treatment Need (IOTN-AC), the Psychosocial Impact of Dental Aesthetics Questionnaire (PIDAQ), and the Oral Health Impact Profile (OHIP-14). Descriptive statistics, Wilcoxon rank-sum tests, Pearson’s chi-squared tests, and Spearman’s correlations were used. Multivariable logistic regression identified predictors of high psychosocial impact (PIDAQ grade). Statistical significance was set at *p* < 0.05.

**Results:**

Of 316 participants (mean age 16.9 ± 3.6 years; 55% male), 45.2% perceived a need for orthodontic treatment (34.8% moderate, 10.4% definite). High psychosocial impact (PIDAQ > 31) was recorded in 63.3% (*n* = 200), with male participants showing significantly higher median PIDAQ scores than females (38 vs. 34, *p* = 0.03). A negative impact on OHRQoL (OHIP-14 > 14) was reported by 32.9% (*n* = 104). IOTN-AC showed mild positive correlations with aesthetic concern (ρ = 0.13) and psychological impact (ρ = 0.18). OHIP-14 was strongly correlated with psychological impact (ρ = 0.62) and social impact (ρ = 0.59). In multivariable analysis, male sex (OR = 2.65, 95% CI:1.48–4.83), higher OHIP-14 score (OR = 1.14, 95% CI:1.10–1.19), and higher IOTN-AC score (OR = 1.30, 95% CI:1.12–1.53) were independent predictors of high psychosocial impact, while tertiary education was protective (OR = 0.07, 95% CI:0.02–0.31).

**Conclusion:**

Self-perceived malocclusion is associated with reduced dental self-confidence, impaired OHRQoL, and adverse social and psychological well-being among Nigerian adolescents and young adults. These associations vary by sex and education level. Early orthodontic assessment and integrated oral health education may mitigate psychosocial burdens.

**Supplementary Information:**

The online version contains supplementary material available at https://doi.org/10.1186/s12903-026-09399-9.

## Introduction

Malocclusion can be defined as irregularities in tooth position, dental arch relationships, or both, affecting approximately 60–70% of the global population [1]. Beyond its functional consequences for mastication, speech, and oral hygiene maintenance, malocclusion carries significant aesthetic implications that can profoundly affect psychosocial well-being, particularly during adolescence and young adulthood, a critical developmental periods for identity formation and social integration [2, 3].

Over recent decades, the understanding of dental aesthetics has evolved from a purely clinical perspective to a biopsychosocial framework that recognizes the interplay between physical appearance, psychological functioning, and social interactions [4]. Dental appearance plays a pivotal role in facial attractiveness perceptions, which in turn influence self-esteem, peer acceptance, and overall quality of life [5, 6].

Approximately 38–45% of adolescents express concerns about their dental appearance, with malocclusion frequently cited as the primary source of dissatisfaction [7]. Previous studies have reported associations between malocclusion and reduced self-esteem, increased social anxiety, depression, and compromised oral health-related quality of life (OHRQoL) [8, 9]. Kragt et al. global systematic review and meta-analysis found a strong correlation between malocclusion severity and psychological distress among adolescents, with effects persisting into young adulthood [10].

However, the relationship between objective malocclusion severity and subjective psychosocial impact is moderated by factors such as cultural beliefs, family attitudes toward dental appearance, personality traits, and access to orthodontic care [11]. Validated instruments including the Psychosocial Impact of Dental Aesthetics Questionnaire (PIDAQ), Oral Health Impact Profile (OHIP), and OHRQoL measures have been developed to capture these patient-reported outcomes [3].

Studies from Brazil [12] and Saudi Arabia [2] have demonstrated negative psychosocial impacts of malocclusion that increase with severity. In sub-Saharan Africa, Tanzanian research by Mtaya et al. correlated malocclusion with perceived orthodontic treatment need and functional limitations [13]. Motloba et al. reported heightened perception of severe malocclusion among young South African adults compared to adolescents [14]. Conversely, Akpasa et al. found no significant effect of malocclusion severity on psychosocial impact among Nigerian adolescents [15]. A study from Birnin Kebbi, Northern Nigeria (Kanmodi et al.), reported that many secondary school students were dissatisfied with their dental aesthetics, consistent with findings from southern Nigeria [16]. However, values attributed to dental aesthetics vary across social and cultural contexts, and data from low-income countries, particularly northern Nigeria, remain limited [16]. This study therefore aimed to evaluate the prevalence and severity of psychosocial burden associated with malocclusion and its correlation with OHRQoL among adolescents and young adults in Kano State, Nigeria.

## Methods

### Study design and setting

This was a cross-sectional study conducted between 13th May 2024–2nd August 2025 among adolescents and young adults aged 13–25 years in Kano metropolis, Nigeria. Participants were recruited from randomly selected secondary schools and Bayero University, Kano.

### Participant recruitment and sampling procedure

A convenience sampling approach was employed to select the study clusters. Secondary schools within Kano metropolis were purposively selected to ensure representation across ownership types (public versus private) and geographical locations (urban versus rural), resulting in the inclusion of eight schools—four public and four private schools. Within each selected school, two arms/classes per grade level (SS1–SS3) were selected based on accessibility. At the tertiary level, six faculties at Bayero University, Kano, were conveniently selected; from each faculty, two departments were chosen, and one lecture cohort per year of study (100–400 L) was included from each department. Eligible students (aged 13–25 years) were identified via official class rosters and enrollment registers. Within the selected clusters, a total enumeration (census) approach was adopted—every student who met the age criterion was invited to participate—thereby minimizing selection bias at the individual level.

### Ethics approval and consent to participate

The study was approved by the Health Research and Ethics Committee of Bayero University, Kano State, Nigeria (NHREC/06/12/19/217). Permission was obtained from the Kano State Ministry of Education, relevant education departments, and school principals. Written informed consent was obtained from all participants aged ≥ 18 years, and from parents/guardians for those aged < 18 years. Written assent was also obtained from all minors using an age-appropriate form approved by the ethics committee. In line with Nigerian guidelines, assent was required for those below 16 years, while participants aged 16–17 years provided assent alongside parental consent. Study details, including risks and benefits, were explained in simple language to ensure understanding. All participants were informed that participation was voluntary and that withdrawal was permitted at any time without consequence.

### Inclusion and exclusion criteria

Participants were eligible for inclusion if they met the following criteria:

- Age: 13–25 years at the time of recruitment;
- Enrollment: Currently registered as a student in one of the participating secondary schools or at Bayero University, Kano; and
- Consent: Willing and able to provide written informed consent (parental/guardian consent for minors, with participant assent).

Students were excluded from the study if they presented with any of the following:

- Orthodontic history: Current or previous orthodontic treatment;
- Craniofacial conditions: Diagnosed craniofacial syndromes or congenital anomalies; or
- Cognitive/communication barriers: Special needs or conditions that would preclude independent comprehension or completion of the self-administered questionnaires.

### Sample size calculation

Sample size was calculated using Cochran’s formula (1977) [17] using N = (Z²pq)/d², where Z = 1.96 for 95% confidence interval, *p* = 38% prevalence from a previous Nigerian study [18], q = 0.62, and d = 0.05 precision. Accounting for a 10% attrition rate, the target sample was 352 participants.

### Recruitment and participant flow

**Fig. 1:**
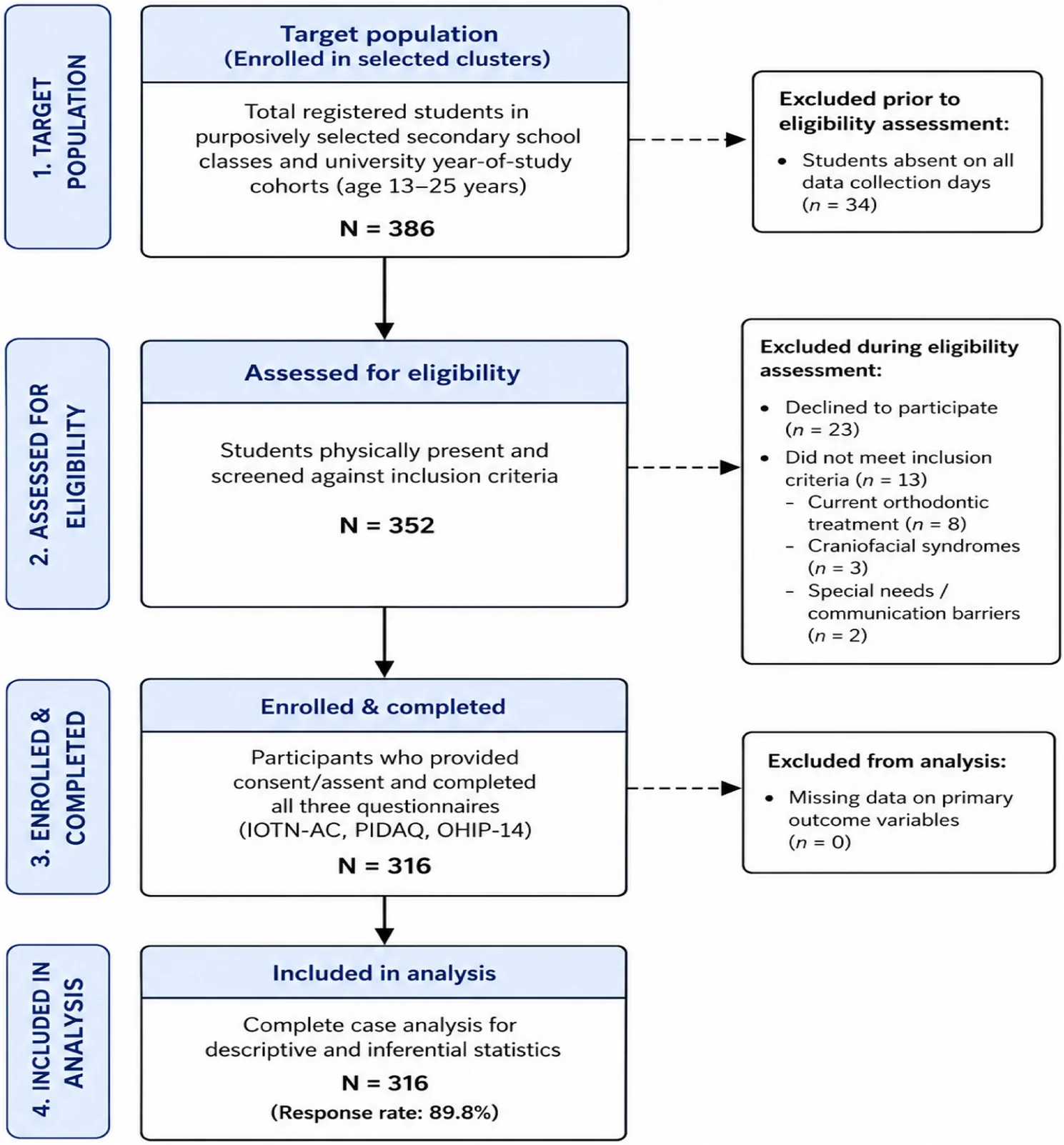
The STROBE flow diagram

The combined registers from all selected clusters identified 386 registered students within the target age range. During data collection, 34 students were absent despite up to three repeated visits, leaving 352 students physically present and assessed for eligibility. Of these, 23 declined to participate, and 13 were excluded for failing to meet the inclusion criteria (8 undergoing active orthodontic treatment, 3 with diagnosed craniofacial syndromes, and 2 with special needs requiring caregiver-assisted communication). Consequently, 316 eligible and consenting students completed the self-administered questionnaires (IOTN-AC, PIDAQ, and OHIP-14), yielding a response rate of 89.8% (316/352) among those assessed and 81.9% (316/386) of the total target population. With no missing data for the primary outcome variables, a complete case analysis was performed, including all 316 participants in the final descriptive and inferential analyses (Fig. 1).

### Variables and measurement instruments

Data were collected using a four-section self-administered questionnaire:

- *Section A (Sociodemographic variables)*: Age, sex, ethnicity, education level (secondary vs. tertiary), and religion.
- *Section B (Psychosocial impact of dental aesthetics)*: The validated PIDAQ, comprising 23 items across four subdomains: dental self-confidence (6 items), aesthetic concern (3 items), psychological impact (7 items), and social impact (7 items). Items are scored on a 5-point Likert scale (0 = not at all, 4 = very much). Total scores range from 0 to 92, with higher scores indicating worse psychosocial impact. A cut-off of 31 was used to classify high vs. low impact as per previous studies [19].
- *Section C (Oral health-related quality of life)*: The validated OHIP-14, consisting of 14 items across seven dimensions: functional limitation, physical pain, psychological discomfort, physical disability, psychological disability, social disability, and handicap. Each item is scored on a 5-point Likert scale (0 = never, 4 = very often). Total scores range from 0 to 56; scores ≤ 14 indicate nil/minimal impact, and scores > 14 indicate negative impact on OHRQoL.
- *Section D (Self-perceived malocclusion)*: The self-rated Aesthetic Component (AC) of the Index of Orthodontic Treatment Need (IOTN-AC), consisting of 10 color-coded photographs showing increasing levels of dental attractiveness (1 = most attractive, 10 = least attractive). Scores 1–4 indicate no perceived treatment need, 5–7 moderate need, and 8–10 definite treatment need.

All questionnaires were administered in English, the medium of instruction in Nigerian schools. Participants completed them in a quiet classroom setting without time limits, with research assistants available for clarification.

### Bias control

To minimize information bias, participants were assured of confidentiality and anonymity, and no identifying information was collected. Questionnaires were coded, and data were stored in a password-protected computer accessible only to the investigators.

### Statistical analysis

All statistical analyses were performed using R and RStudio (Version 2023.06.1 + 524). Normality of continuous variables was assessed using the Shapiro–Wilk test, and homogeneity of variances was evaluated using Levene’s test. As most continuous variables deviated from a normal distribution, non-parametric methods were applied for all bivariate comparisons and correlation analyses. Continuous variables were summarized as means with standard deviations for normally distributed data and medians with interquartile ranges (IQR) for non-normally distributed data. Categorical variables were presented as frequencies and percentages.

Between-group comparisons of non-normally distributed continuous variables were performed using the Wilcoxon rank-sum (Mann–Whitney U) test for two-group comparisons and the Kruskal–Wallis test for comparisons involving three or more groups; Mood’s median test was used as a supplementary non-parametric alternative where appropriate. Associations between categorical variables were assessed using Pearson’s chi-squared test, or Fisher’s exact test where expected cell counts were less than 5. Spearman’s rank correlation coefficient (ρ) was calculated to evaluate the strength and direction of associations among the IOTN-AC, OHIP-14, and PIDAQ subdomain scores. To determine whether the effect of ethnicity on psychosocial impact differs by age—and to account for potential confounding where ethnic differences may reflect age-related developmental or educational disparities—an age–ethnicity interaction analysis was performed. This approach allowed us to assess whether the observed ethnic differences in psychosocial outcomes were independent of age or partly attributable to age-related factors.

Multivariable logistic regression with a bootstrapped stepwise selection procedure (1000 resamples) was performed to identify independent predictors of high psychosocial impact. The dependent variable was the PIDAQ grade, dichotomized as “high” versus “low” using a median split of the total PIDAQ score (scores above the median were classified as high). Independent variables entered into the initial model included age, sex, education level, OHIP-14 total score, and IOTN-AC score. Ethnicity was not retained in the final model following the stepwise selection procedure. Adjusted odds ratios (OR) with 95% confidence intervals (CI) were computed for all retained predictors. Model calibration was assessed using the Hosmer–Lemeshow goodness-of-fit test (*p* > 0.05 indicating adequate fit), and overall explanatory power was evaluated using Nagelkerke’s pseudo-R². Multicollinearity among the retained variables was examined using the variance inflation factor (VIF), with a threshold of VIF < 5 indicating no significant collinearity. All statistical tests were two-tailed, and a p-value < 0.05 was considered statistically significant.

## Results

### Participant characteristics

A total of 316 participants were enrolled. The mean age was 16.9 ± 3.6 years (range 13–30 years). Males comprised 55.0% (*n* = 175). Hausa ethnicity accounted for 76.9% (*n* = 243), followed by other ethnic groups (12.0%, *n* = 38) and Fulani (11.1%, *n* = 35). Most participants (71.8%, *n* = 227) were secondary school students, and 96% (*n* = 303) were Muslim (Table 1).

**Fig. 2:**
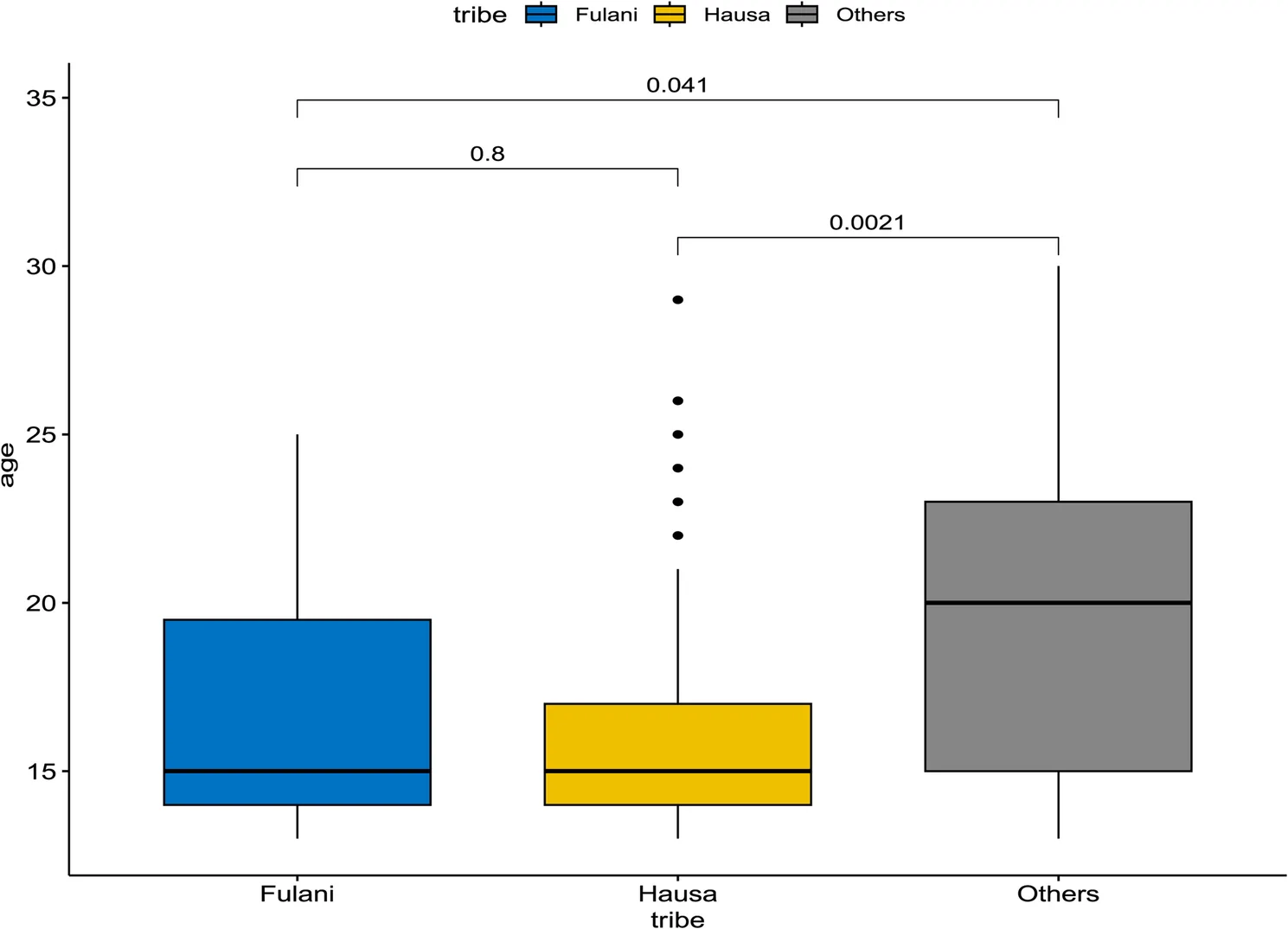
Age distribution by ethnicity. Significant differences were observed across ethnic groups (Kruskal–Wallis *p* = 0.008), driven by pairwise differences between Hausa vs. “other” (*p* = 0.0021) and Fulani vs. “other” (*p* = 0.041)

Males were significantly older than females (mean 17.3 ± 4.2 vs. 16.4 ± 3.8 years; mean difference: 0.9 years, 95% CI: 0.4–1.4; *p* < 0.001). Age also differed significantly by ethnicity (*p* = 0.008; Fig. 2); participants from “other” ethnic groups were older (mean 19.2 years, 95% CI: 17.1–21.3) than Hausa (mean 16.6 years, 95% CI: 16.2–17.0) and Fulani participants (mean 16.8 years, 95% CI: 15.5–18.1). Pairwise post-hoc comparisons showed that the difference between Hausa and “other” ethnic groups was statistically significant (*p* = 0.002), as was the difference between Fulani and “other” groups (*p* = 0.04).

**Table 1:** Sociodemographic characteristics of the study participants (*N* = 316)

Variable	*N* (%) or Mean (SD)
Age, years	16.9 (3.6)
Sex	
Female	141 (45.0)
Male	175 (55.0)
Ethnicity	
Hausa	243 (76.9)
Fulani	35 (11.1)
Others^a^	38 (12.0)
Education level	
Secondary	227 (71.8)
Tertiary	89 (28.2)

Abbreviation: *SD* standard deviation

^a^Others include Igbo, Yoruba, Igala, Kanuri, and Shua

### Self-perceived malocclusion (IOTN-AC)

Overall, 54.7% (*n* = 173) perceived no need for orthodontic treatment (IOTN-AC grades 1–4), 34.8% (*n* = 110) perceived moderate need (grades 5–7), and 10.4% (*n* = 33) perceived definite need (grades 8–10). Perception did not differ significantly by age (*p* = 0.20) or sex (*p* = 0.50) but varied significantly by ethnicity (*p* = 0.026) and education level (*p* < 0.001). Post-hoc analysis showed that the ethnic difference was primarily between Fulani and Hausa participants (*p* = 0.03) (Table 2).

**Table 2:** Distribution of self-perceived orthodontic treatment need (IOTN–AC) according to sociodemographic characteristics

Characteristic	No need (grades 1–4) (*n* = 173)	Moderate need (grades 5–7) (*n* = 110)	Definite need (grades 8–10) (*n* = 33)	*P* value
Age, years, median (IQR)	15.0 (14.0–17.0)	16.0 (14.0–21.0)	15.0 (15.0–16.0)	0.20
Sex				0.50
Female	81 (47.0)	48 (44.0)	12 (36.0)	
Male	92 (53.0)	62 (56.0)	21 (64.0)	
Ethnicity				0.026^a^
Fulani	26 (15.0)	9 (8.2)	0 (0.0)	
Hausa	128 (74.0)	84 (76.0)	31 (94.0)	
Others^b^	19 (11.0)	17 (15.0)	2 (6.1)	
Education level				< 0.001
Secondary	132 (76.0)	65 (59.0)	30 (90.9)	
Tertiary	41 (24.0)	45 (41.0)	3 (9.1)	

Abbreviations: *IOTN–AC* Index of Orthodontic Treatment Need–Aesthetic Component, *IQR* interquartile range

^a^*P* values were obtained using the Kruskal–Wallis test for age and the chi-squared test for categorical variables. Post hoc pairwise comparison showed a significant difference between Fulani and Hausa participants (*P* = 0.026). ^b^Others include Igbo, Yoruba, Igala, Kanuri, and Shua

### Oral health-related quality of life (OHIP-14)

**Fig. 3:**
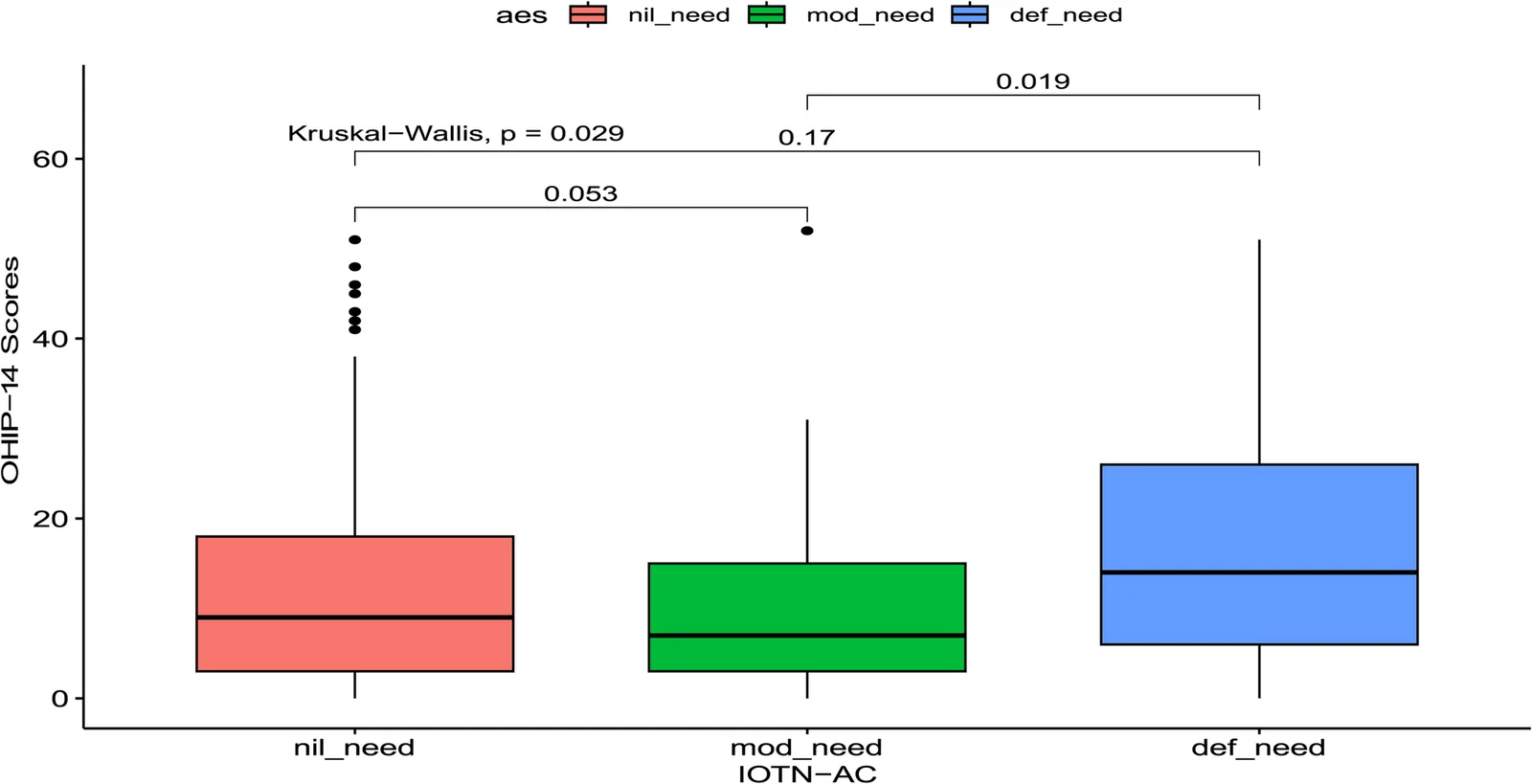
Boxplot of OHIP-14 scores across IOTN-AC grades showing median (horizontal line) and IQR (boxes). Scores increased significantly with greater treatment need (Kruskal–Wallis *p* = 0.029), with definite need scores significantly higher than moderate need (*p* = 0.019)

The median OHIP-14 score was 9 (range 0–52; IQR: 4–18). Using a cut-off score of 15, 67.1% (*n* = 212) reported nil or minimal impact on OHRQoL, while 32.9% (*n* = 104) reported a negative impact. Negative impact was more common among younger participants (median age: 15 years vs. 16 years; median difference: 1 year, 95% CI: 0.5–1.5; *p* < 0.001) and was disproportionately observed among secondary school students (86.5% of those with negative impact were in secondary school; 95% CI: 78.5–92.4; *p* < 0.001). No significant associations were observed with sex (*p* = 0.3) or ethnicity (*p* = 0.5). OHIP-14 scores increased with greater treatment need: participants with no need had a median score of 7 (IQR: 3–15), those with moderate need had a median of 7 (IQR: 4–14), and those with definite need had a median of 14 (IQR: 8–25) (*p* = 0.029; Fig. 3).

### Psychosocial impact (PIDAQ)

The median total PIDAQ score was 37 (IQR: 27–49; range: 1–85). Using a cut-off score of 31, 63.3% (*n* = 200) were classified as having a high PIDAQ grade (indicating negative psychosocial impact). PIDAQ scores were significantly higher among males (median: 38 vs. 34; median difference: 4, 95% CI: 1–7; *p* = 0.008), Hausa participants (median: 39 vs. 30 for Fulani; median difference: 9, 95% CI: 4–14; *p* = 0.003), secondary school students (median: 42 vs. 28; median difference: 14, 95% CI: 9–19; *p* < 0.001), and those with definite treatment need (median: 48 vs. 35 for no need; median difference: 13, 95% CI: 6–20; *p* = 0.003) (Table 3).

**Table 3:** Psychosocial impact of malocclusion by sociodemographic and clinical characteristics

Characteristic	High impact (PIDAQ > 31) (*n* = 200)	Low impact (PIDAQ ≤ 31) (*n* = 116)	*P* value	Effect estimate (95% CI)
Age, years, median (IQR)	15.0 (14.0–16.0)	17.5 (14.8–21.0)	< 0.001	Median difference: 2.5 years (1.8–3.2)
Sex			0.008	
Female	78 (39.0)	63 (54.0)		Reference
Male	122 (61.0)	53 (46.0)		OR 1.86 (1.18–2.94)
Ethnicity			0.007	
Fulani	14 (7.0)	21 (18.0)		Reference
Hausa	166 (83.0)	77 (66.0)		OR 3.23 (1.56–6.68)
Othersᵃ	20 (10.0)	18 (16.0)		OR 1.67 (0.69–4.04)
Education level			< 0.001	
Secondary	168 (84.0)	59 (51.0)		Reference
Tertiary	32 (16.0)	57 (49.0)		OR 0.20 (0.12–0.34)
Aesthetic perception (IOTN–AC)			0.003	
No need (grades 1–4)	102 (51.0)	71 (61.0)		Reference
Moderate need (grades 5–7)	69 (35.0)	41 (35.0)		OR 1.17 (0.72–1.90)
Definite need (grades 8–10)	29 (15.0)	4 (3.4)		OR 5.04 (1.70–14.96)

*Abbreviations*: *PIDAQ* Psychosocial Impact of Dental Aesthetics Questionnaire, *IQR* interquartile range, *OR* odds ratio, *CI* confidence interval

ᵃOthers include Igbo, Yoruba, Igala, Kanuri, and Shua. P values were obtained using the Mann–Whitney U test for age and the chi-squared test for categorical variables while Odds ratios (95% CIs) were estimated using univariate logistic regression

### Correlations among self-perceived malocclusion severity (IOTN-AC), oral health-related quality of life (OHIP-14), and psychosocial impact domains (PIDAQ)

Spearman’s correlation analysis revealed that self-perceived malocclusion severity (IOTN-AC) demonstrated only weak positive correlations with aesthetic concern (ρ = 0.13) and psychological impact (ρ = 0.18), indicating that perceived severity alone is a poor predictor of psychosocial burden. In contrast, oral health-related quality of life (OHIP-14) exhibited substantially stronger associations, with strong positive correlations with psychological impact (ρ = 0.62) and social impact (ρ = 0.59), a moderate positive correlation with aesthetic concern (ρ = 0.42), and a weak negative correlation with dental self-confidence (ρ = − 0.32). These findings suggest that functional impairment and daily quality of life are more centrally linked to psychosocial distress than aesthetic severity alone.

**Fig. 4:**
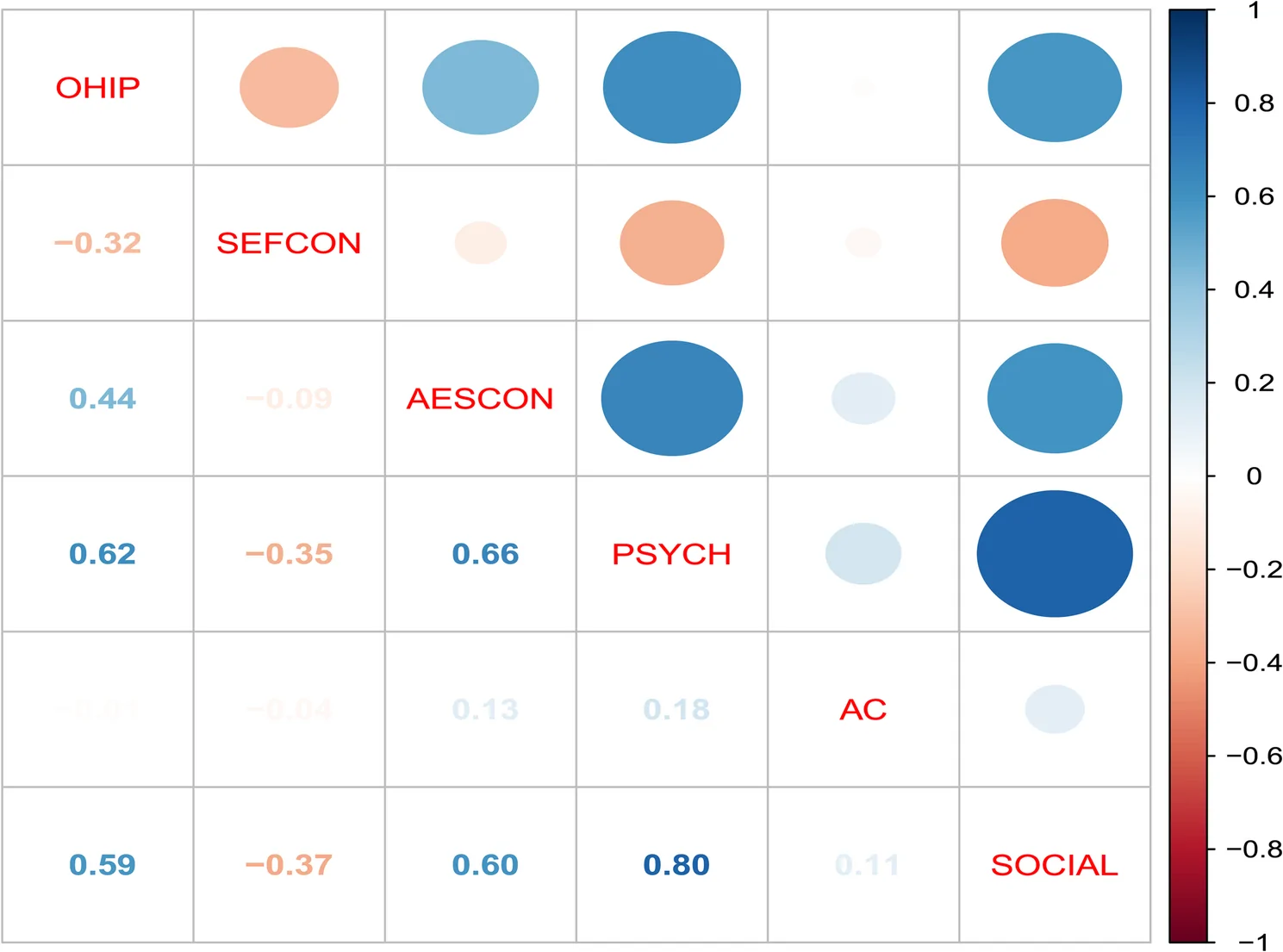
Spearman’s correlation heatmap of IOTN-AC, OHIP-14, and PIDAQ subdomains (SEFCON, AESCON, PSYCH, SOCIAL). Darker shades indicate stronger correlations (blue positive, red negative); all |ρ| ≥ 0.13 were significant (*p* < 0.05), with 95% CIs shown in parentheses

Within the psychosocial impact domains, aesthetic concern, psychological impact, and social impact were strongly positively intercorrelated (ρ = 0.60–0.80), indicating that these dimensions are mutually reinforcing. Conversely, dental self-confidence showed moderate negative correlations with aesthetic concern (ρ = − 0.35), psychological impact (ρ = − 0.37), and social impact (ρ = − 0.44), suggesting that confidence in one’s smile serves as a protective factor against psychosocial burden. Collectively, these findings underscore that functional impairment is more strongly associated with psychosocial outcomes than aesthetic severity, and that dental self-confidence may buffer against the negative psychosocial consequences of malocclusion. These correlations are shown in Fig. 4.

### Multivariable logistic regression analysis of predictors of high psychosocial impact

A stepwise logistic regression model was constructed to identify independent predictors of high psychosocial impact (PIDAQ grade > 31). The final model demonstrated good fit (Hosmer–Lemeshow test: *p* = 0.32; Nagelkerke pseudo-R² = 0.41). Male sex emerged as a significant independent predictor, with males having 2.65 times the odds of experiencing high psychosocial impact compared to females (OR = 2.65; 95% CI: 1.48–4.83; *p* = 0.001). Both oral health-related quality of life and self-perceived malocclusion severity were also independently associated with psychosocial burden (Table 4).

Each unit increase in OHIP-14 score was associated with a 14% increase in the odds of high psychosocial impact (OR = 1.14; 95% CI: 1.10–1.19; *p* < 0.001), while each unit increase in IOTN-AC score corresponded to a 30% increase in odds (OR = 1.30; 95% CI: 1.12–1.53; *p* < 0.001). In contrast, tertiary education exerted a strong protective effect, reducing the odds of high psychosocial impact by approximately 93% compared to secondary education (OR = 0.07; 95% CI: 0.02–0.31; *p* < 0.001). Age was not retained as a statistically significant predictor in the final model (OR = 1.18; 95% CI: 0.98–1.43; *p* = 0.078).

**Table 4:** Multivariable logistic regression analysis of predictors of high psychosocial impact (PIDAQ score > 31)

Predictor	Adjusted OR	95% CI	*P* value
Age (per year)	1.18	0.98–1.43	0.078
Sex (reference: Female)			
Male	2.65	1.48–4.83	0.001
Education level (reference: Secondary)			
Tertiary	0.07	0.02–0.31	< 0.001
OHIP-14 total score (per unit)	1.14	1.10–1.19	< 0.001
IOTN–AC score (per unit)	1.30	1.12–1.53	< 0.001

Abbreviations: *PIDAQ* Psychosocial Impact of Dental Aesthetics Questionnaire, *OR* odds ratio, *CI* confidence interval, *OHIP-14* Oral Health Impact Profile-14, *IOTN–AC* Index of Orthodontic Treatment Need–Aesthetic Component

Model fit: Hosmer–Lemeshow goodness-of-fit test, *P* = 0.32; Nagelkerke pseudo-R² = 0.41

## Discussion

This cross-sectional study of 316 Nigerian adolescents and young adults found that nearly two-thirds experienced high psychosocial impact from self-perceived malocclusion, and one-third reported impaired oral health-related quality of life. Male gender, lower educational level, poorer OHRQoL, and greater self-perceived malocclusion severity independently predicted higher psychosocial burden [10, 13]. These findings contribute to the growing body of evidence that malocclusion extends beyond a purely functional or aesthetic concern to encompass significant psychological and social consequences, particularly in culturally diverse and resource-limited settings.

The high prevalence of psychosocial impact observed in our study aligns closely with findings from previous Nigerian studies. Kanmodi et al. [15, 16] and Isiekwe et al. [20] reported that a substantial proportion of secondary school students in Northern and Southern Nigeria, respectively, were dissatisfied with their dental aesthetic appearance, while a recent study from South-South Nigeria (Etim & Umeh) documented mean PIDAQ scores comparable to those in our cohort [21]. Our findings are also consistent with international reports, including a Malaysian study that found the vast majority of young adults experienced psychosocial impact from dental aesthetics, with those who perceived themselves as having malocclusion reporting substantially greater impact than those who did not [22].

The male predominance observed in our study warrants particular attention. While studies from Brazil [23], Turkey [24], and Malaysia [22] have reported greater impact among females, our finding contrasts with these international patterns. However, it is consistent with Motloba et al. [14], who reported that malocclusion, particularly when self-perceived, has a significant negative impact on psychological wellbeing, especially among older and male patients. Similarly, a study on Spanish adolescents found that gender was a predictive variable for psychological impact [9]. The male predominance in our Northern Nigerian cohort may reflect cultural and geographical factors unique to this region, where societal expectations and peer scrutiny of appearance may be particularly intense for adolescent males. This underscores the need for culturally contextualized interpretations of psychosocial impact, as gender-based differences appear to be population-specific rather than universal.

The proportion of participants who perceived a need for orthodontic treatment in our study was comparable to an earlier Nigerian report [18] but lower than global estimates of objective malocclusion [1]. This discrepancy highlights that self-perceived need does not always align with clinical severity, a phenomenon previously documented in the literature [25, 26]. Indeed, Doughan et al. [25] found that the aesthetic component of the IOTN was better associated with the psychosocial implications of malocclusion than objective indices alone, and that the vast majority of adolescents wished their teeth looked better, with many unhappy about their smile aesthetics. This reinforces the notion that patient-centered perceptions of dental aesthetics often diverge from clinician-assessed normative need [26].

The modest association between self-perceived malocclusion and psychosocial impact in our study suggests that while malocclusion contributes to psychosocial burden, other factors—such as personality traits, social support, and cultural norms—play substantial roles. This is consistent with the literature: studies from India [27, 28] have reported positive associations between aesthetic indices and psychosocial impact, with some demonstrating strong convergent validity. The relatively modest strength of association in our sample may reflect the influence of unmeasured psychosocial mediators that buffer or amplify the impact of objectively assessed malocclusion [25].

One-third of participants reported negative impact on oral health-related quality of life, similar to findings from previous Nigerian studies and international research. A recent study on young adults found that OHRQoL measures, along with other variables, collectively explained a substantial proportion of the variance in psychosocial impact scores [26]. Korean research similarly reported a positive association between OHRQoL and psychosocial impact, with women experiencing greater impact than men [29].

The strong associations observed in our study between OHRQoL and both psychological and social impact indicate that malocclusion affects not only functional and physical domains but also emotional and social well-being. This aligns with the biopsychosocial model [3] and is supported by a growing evidence base demonstrating that malocclusion influences psychological functioning, self-confidence, and body image [10]. Furthermore, patients with more severe malocclusion have been shown to report poorer self-rated dental appearance and OHRQoL associated with functional limitations, psychological discomfort, and physical disability persisting into adulthood [10, 13]. These findings suggest that interventions should address functional and aesthetic concerns concurrently, as the psychological burden of malocclusion is not merely a byproduct of physical impairment but a distinct dimension of patient experience.

Multivariable analysis identified male gender, lower education, poorer OHRQoL, and greater self-perceived malocclusion severity as independent predictors of high psychosocial impact. The finding that aesthetic self-perception independently predicts psychosocial burden is corroborated by recent studies demonstrating that the aesthetic component of the IOTN is better associated with psychosocial implications than other occlusal indices [25], with strong convergent validity reported in psychometric evaluations.

The strong protective effect of tertiary education is particularly noteworthy. University students may have greater health literacy, coping mechanisms, or access to information that mitigates psychosocial distress. Studies from Nigeria have demonstrated that higher education correlates with increased health information seeking and healthier behaviors, although formal education alone does not guarantee optimal health literacy or service utilization [30, 31]. Alternatively, university students may have already sought corrective treatment (though current orthodontic treatment was an exclusion criterion, prior treatment was not), or they may have developed greater acceptance of their appearance through exposure to diverse social environments. De Paula et al. similarly found that aesthetic perception and OHRQoL were strong predictors of psychosocial impact [19]. Interestingly, a study on Nigerian adolescents found that severity of malocclusion as measured by the DAI had no significant effect on psychosocial impact, and total psychosocial impact scores showed no significant differences by age, gender, or academic level—a finding that contrasts with our results and underscores the importance of context-specific investigations [15].

### Strengths and limitations

Strengths of this study include the use of validated, internationally recognized instruments (IOTN-AC, PIDAQ, OHIP-14), a sample spanning both secondary and tertiary education settings, and adherence to STROBE guidelines for cross-sectional studies.

Several limitations must be acknowledged. First, the convenience sampling method may limit generalizability beyond Kano metropolis. Second, the cross-sectional design precludes causal inferences. Third, we relied on self-reported malocclusion (IOTN-AC) rather than clinical examination, which may have underestimated severity and introduced self-report bias. However, self-perception is arguably more relevant to psychosocial outcomes than objective severity, as supported by Motloba et al. [14] who concluded that malocclusion, particularly when self-perceived, has a significant negative impact on psychological wellbeing, and by research demonstrating significant differences between professional views and patient perceptions of dental appearance and orthodontic treatment need. Fourth, the 30-year-old outlier (*n* = 1) may slightly skew age-related findings, though sensitivity analysis excluding this participant did not change conclusions. Fifth, the PIDAQ cut-off of 31, while previously used [19], has not been formally validated in Nigerian populations. Finally, unmeasured confounders such as personality traits, socioeconomic status, and family attitudes toward dental appearance may influence the observed associations. Indeed, the literature suggests that psychological traits demonstrate statistically significant associations with malocclusion severity and patient self-motivation for treatment, highlighting the need for future research to account for these complex interrelationships.

### Implications for practice and research

These findings suggest that orthodontic services in low-resource settings should not be viewed solely as cosmetic interventions but as potential contributors to mental health and quality of life. The psychosocial and aesthetic impacts of malocclusion have been shown to prevail over oral and functional problems [26], and PIDAQ has been demonstrated to discriminate better than generic OHRQoL measures between varying degrees of dental aesthetics [26]. Periodic school-based oral health programs should include education on the psychosocial aspects of malocclusion and available treatment options. Given that orthodontic patients with higher age and being female experience greater negative psychosocial impact from malocclusion—though our study found male predominance, highlighting population-specific variation—tailored interventions that consider demographic and cultural factors are warranted. For researchers, longitudinal studies incorporating clinical assessments and validated culturally adapted instruments are needed to establish temporality and explore moderating factors.

## Conclusion

Self-perceived malocclusion is associated with significant psychosocial burden and impaired oral health-related quality of life among Nigerian adolescents and young adults, particularly among males and secondary school students. These findings support the integration of psychosocial assessment into routine dental care and the development of school-based oral health promotion programs.

## Recommendations

1. Early orthodontic assessment during adolescence: Encourage early orthodontic evaluation during adolescence to enable timely identification and intervention, thereby mitigating long-term psychosocial burden.
2. School-based oral health education: Integrate education on the aesthetic and functional consequences of malocclusion into periodic school visits to improve awareness, reduce stigma, and promote help-seeking behaviors.
3. Future research directions: Employ clinical examinations alongside patient-reported outcomes, and explore culturally tailored interventions that account for ethnic, social, and contextual factors influencing perceptions of dental aesthetics and treatment needs.
4. Address gender disparities in psychosocial impact: Given the male predominance of high psychosocial impact observed in this study, targeted interventions for adolescent males—particularly in culturally conservative settings—should be developed. These interventions may include male-focused oral health promotion campaigns, peer support programs, and culturally sensitive counseling to address appearance-related concerns.

## Supplementary Information

Supplementary Material 1.

## Data Availability

The datasets used and/or analyzed during the current study are available from the corresponding author on reasonable request.

## Abbreviations

AC: Aesthetic Component
IOTN: Index of Orthodontic Treatment Need
OHIP-14: Oral Health Impact Profile-14
OHRQoL: Oral Health-Related Quality of Life
PIDAQ: Psychosocial Impact of Dental Aesthetics Questionnaire
STROBE: Strengthening the Reporting of Observational Studies in Epidemiology
