# Supplementary material for: Psychosocial impact of self-perceived malocclusion on oral health-related quality of life in Nigerian adolescents and young adults: a cross-sectional study
Source: BMC Oral Health. 2026 Jul 24;26:2080. doi: 10.1186/s12903-026-09399-9 (PMC13689316; doi:10.1186/s12903-026-09399-9)
Supplement: Supplementary file 1 [file 12903_2026_9399_MOESM1_ESM.docx]

**Supplementary Table S1. STROBE checklist for cross-sectional studies**

Checklist of items addressed in the manuscript according to the STROBE Statement.

| Section & Item | Item No. | Recommendation | Where reported in manuscript |
| --- | --- | --- | --- |
| Title and abstract | 1a | Indicate study design in title/abstract | Title and Abstract |
| Title and abstract | 1b | Provide balanced abstract summary | Abstract sections |
| Background/rationale | 2 | Explain scientific background and rationale | Introduction paragraphs 1–4 |
| Objectives | 3 | State specific objectives and hypotheses | Introduction final paragraph |
| Study design | 4 | Present key elements of study design | Methods – Study design and setting |
| Setting | 5 | Describe setting, locations, and dates | Methods – Study design and setting |
| Participants | 6a | Describe eligibility criteria and participant selection | Methods – Participants |
| Participants | 6b | Report participation and missing observations | Methods – Sample size |
| Variables | 7 | Define outcomes, predictors, and confounders | Methods – Variables and measurement instruments |
| Data sources/measurement | 8 | Describe measurements and assessments | Methods – Variables and measurement instruments |
| Bias | 9 | Describe bias reduction methods | Methods – Bias control |
| Study size | 10 | Explain sample size determination | Methods – Sample size |
| Quantitative variables | 11 | Explain handling of quantitative variables | Methods – Statistical analysis |
| Statistical methods | 12a | Describe statistical methods | Methods – Statistical analysis |
| Statistical methods | 12b | Describe subgroup analyses | Tables 2 and 3 |
| Statistical methods | 12c | Explain handling of missing data | Complete-case analysis implied |
| Statistical methods | 12d | Describe analytical methods for sampling strategy | Convenience sampling described |
| Participants | 13a | Report participant numbers at each stage | Results – Participant characteristics |
| Participants | 13b | Explain non-participation | Response rate described |
| Participants | 13c | Consider use of flow diagram | Figure 1 |
| Descriptive data | 14a | Describe participant characteristics | Tables 1 and 2 |
| Descriptive data | 14b | Indicate missing data | Zero |
| Outcome data | 15 | Report outcome events and summary measures | Results and Table 3 |
| Main results | 16a | Provide estimates and precision | Table 4 |
| Main results | 16b | Report category boundaries | Methods section |
| Main results | 16c | Translate risk estimates if relevant | Not applicable |
| Other analyses | 17 | Report subgroup or sensitivity analyses | Tables 2 and 3 |
| Key results | 18 | Summarize key findings | Discussion |
| Limitations | 19 | Discuss limitations and bias | Discussion – Strengths and limitations |
| Interpretation | 20 | Provide overall interpretation | Discussion and Conclusion |
| Generalisability | 21 | Discuss external validity | Discussion |
| Funding | 22 | Describe funding source | Declarations – Funding |

Abbreviation: STROBE, Strengthening the Reporting of Observational Studies in Epidemiology.
